# The F-box protein Cdc4/Fbxw7 is a novel regulator of neural crest development in *Xenopus laevis*

**DOI:** 10.1186/1749-8104-5-1

**Published:** 2010-01-04

**Authors:** Alexandra D Almeida, Helen M Wise, Christopher J Hindley, Michael K Slevin, Rebecca S Hartley, Anna Philpott

**Affiliations:** 1 Department of Oncology, University of Cambridge, Hutchison-MRC Research Centre, Addenbrookes Hospital, Hills Road, Cambridge, CB2 0XZ, UK; 2 Current address: Division of Virology, Department of Pathology, University of Cambridge, Tennis Court Road, CB2 1QP, UK; 3 Program in Molecular Biology and Biotechnology, University of North Carolina at Chapel Hill, Chapel Hill, NC 27599, USA; 4 Department of Cell Biology and Physiology, and Cancer Center, University of New Mexico Health Sciences Center, Albuquerque, NM 87131, USA

## Abstract

**Background:**

The neural crest is a unique population of cells that arise in the vertebrate ectoderm at the neural plate border after which they migrate extensively throughout the embryo, giving rise to a wide range of derivatives. A number of proteins involved in neural crest development have dynamic expression patterns, and it is becoming clear that ubiquitin-mediated protein degradation is partly responsible for this.

**Results:**

Here we demonstrate a novel role for the F-box protein Cdc4/Fbxw7 in neural crest development. Two isoforms of *Xenopus laevis *Cdc4 were identified, and designated xCdc4α and xCdc4β. These are highly conserved with vertebrate Cdc4 orthologs, and the *Xenopus *proteins are functionally equivalent in terms of their ability to degrade Cyclin E, an established vertebrate Cdc4 target. Blocking xCdc4 function specifically inhibited neural crest development at an early stage, prior to expression of *c-Myc*, *Snail2 *and *Snail*.

**Conclusions:**

We demonstrate that Cdc4, an ubiquitin E3 ligase subunit previously identified as targeting primarily cell cycle regulators for proteolysis, has additional roles in control of formation of the neural crest. Hence, we identify Cdc4 as a protein with separable but complementary functions in control of cell proliferation and differentiation.

## Background

During the development of multi-cellular organisms, cells receive signals and must elicit the appropriate response. This involves changes in the level and activity of proteins, and targeted proteolysis represents a rapid and irreversible mechanism to block protein function. During regulated proteolysis, proteins are targeted for degradation by covalent attachment of the 76 amino acid protein ubiquitin, and the polyubiquitin chains assembled on the target protein serve as signals for degradation by the 26S proteasome. Transfer of ubiquitin onto target proteins is catalyzed by a hierarchical multi-enzyme cascade. An E1 (ubiquitin activating) enzyme forms a thioester linkage with the carboxyl terminus of ubiquitin, in an ATP-dependent process. Ubiquitin is then transferred to an E2 (ubiquitin conjugating) enzyme. E3 (ubiquitin ligase) enzymes recruit distinct substrates, allowing ubiquitin transfer, and confer specificity on the ubiquitin proteasome system.

RING (Really Interesting New Gene) E3s are the largest class of E3 ligases, and the human genome encodes approximately 400 proteins with a RING domain [1]. Conserved cysteines and histidines coordinate two zinc ions in the RING domain, which is important for the recruitment and activation of E2 enzymes. Skp1-Cullin1-F-box (SCF) E3 ligases are a large class of modular RING E3 ligases that have the RING component Roc1 (also known as Rbx1 and Hrt1). Cullin1 forms a scaffold to recruit the E2 (via Roc1) and the F-box protein (via binding of the F-box to Skp1) [2,3]. The F-box component of these E3 ligases is variable, and different F-box proteins recruit different substrates via carboxy-terminal domains, allowing SCF ligases to target a huge number of substrates [4].

Cdc4 (also known as Fbw7), one of the most extensively studied F-box proteins, was originally identified in *Saccharomyces cerevisiae*, where it was shown to degrade the cyclin-dependent kinase inhibitor Sic1 [3-8]. In mammals, there are three isoforms of Cdc4: alpha (α), beta (β) and gamma (γ). These are produced by alternative splicing of three unique 5' exons to ten common 3' exons, such that the resulting proteins differ only at their amino termini [9,10]. In mammals, known Cdc4 substrates include c-Myc, c-Jun, Cyclin E, Notch intracellular domain, c-Myb, sterol regulatory element binding proteins (SREBPs) and steroid receptor coactivator-3 (SRC3) [9,11-15]. Given these substrates, it is perhaps unsurprising that Cdc4 has been shown to be a haplo-insufficient tumor suppressor gene [16]. This list of substrates also suggests that Cdc4 could regulate developmental events, and attempts to generate knock-out mice led to an embryonic lethal phenotype [17]. We became interested in a role for Cdc4 during neural crest development in particular because several of its substrates have been implicated in the development of this tissue, for example, c-Myc and Notch intracellular domain [18,19].

The neural crest is a unique population of cells, arising at the neural plate border in response to bone morphogenetic protein, Wnt and fibroblast growth factor signaling (for reviews, see [20,21]). Neural crest cells are initially multipotent, but subsequently undergo an epithelial to mesenchymal transition and migrate throughout the embryo, where they give rise to a wide range of derivatives (for reviews, see [22,23]). These include the neurons and glia of the peripheral nervous system, the autonomic nervous system, cartilage, bone, connective tissue, cardiac cells and melanocytes. The induction of the neural crest is often defined according to the expression of neural crest specifier genes, including the transcriptional repressors *Snail2 *and *Snail *[24,25]. A number of proteins involved in neural crest development display dynamic expression patterns, and it is becoming apparent that several are targets of the ubiquitin proteasome system. For example, Snail2 is degraded by the F-box protein Partner of paired (Ppa) [26].

Here we describe identification of the *Xenopus laevis *homologues of Cdc4, which are highly conserved at the sequence level, and are also functionally equivalent in terms of their ability to degrade Cyclin E. Two isoforms of *Xenopus *Cdc4 (xCdc4α and xCdc4β) are found to be dynamically expressed throughout early *Xenopus *development, with particular enrichment in neural crest and neural crest-derived tissues. Inhibition of xCdc4 activity, using dominant negative F-box mutants, blocks neural crest development, without affecting cell division or cell survival, nor affecting development of the other tissues in which they are expressed. Thus, Cdc4 directly and specifically regulates neural crest formation, independent of a previously described ability to regulate the cell cycle.

## Results

### *X. laevis *encodes two isoforms of Cdc4: xCdc4α and xCdc4β

The pseudotetraploid genome of *X. laevis *presents unique challenges to identifying genes reported in other model systems. In contrast, *Xenopus tropicalis *is possessed of a diploid genome, making it well suited for genetic manipulation and bioinformatic analysis. The existence of *X. tropicalis *and *X. laevis *in the same genus - therefore sharing a high level of evolutionary conservation between their respective genes - suggested to us a method of harnessing the sequenced genome of *X. tropicalis *to identify potential orthologs of human Cdc4 (hCdc4) present in *X. laevis*. BLAST of hCdc4α (GenBank accession number AY049984) against the *X. tropicalis *genome revealed a sequence on scaffold 60:1694203-1694241 with strong nucleotide complementarity to the first exon of hCdc4α. Further downstream (scaffold 60:1696322-1,729,489) were exons 2 to 11, whose sequence corresponded to the conserved regions found within hCdc4.

PCR primers targeting the identified xCdc4 sequence were used on oligo d(T) primed mRNA derived from stage 20 *X. laevis *embryos. We performed 5' and 3' rapid amplification of cDNA ends (RACE) to isolate a full length product. Although slightly truncated, the protein was most similar to the β isoform of hCdc4 and has been designated xCdc4β (Additional file 1). Analysis of the aligned regions demonstrates that xCdc4β was 98% identical at the amino acid level and 83% identical at the nucleotide level to hCdc4β. In comparison to other *X. laevis *F-box proteins, xCdc4β was 27% identical and 43% similar to β -TRCP (β -Transducin repeat containing protein; GenBank accession number M98268) [27], and 17% identical and 20% similar to Skp2 (GenBank accession number DQ228920) [28].

A second isoform of xCdc4 was similarly cloned from stage 7 *X. laevis *embryos (GenBank accession number DQ666345). Exon 1 was found on scaffold 60:1646293-1646793. Analysis demonstrated nearly perfect conservation at the amino acid level to the common set of exons, 2 to 11, that occur in hCdc4α and hCdc4β (data not shown). Additionally, the second identified xCdc4 contained an amino terminal exon most similar to the one present in human Cdc4α and is referred to hereafter as xCdc4α. Furthermore, exon 1 of xCdc4β is spliced to the same downstream exons, 2 to 11, used by xCdc4α. Alignment of their amino acid sequences showed strong conservation; differences in amino acid identity being conserved by use of residues with similar characteristics. Furthermore, both the isoform specific nuclear localization signal (amino acids 11 to 14) and common nuclear localization signal (amino acids 169 to 172) found in hCdc4α are present in xCdc4α, which is suggestive of a similar pattern of localization and regulation [29].

From the region of the gene corresponding to the second exon, xCdc4α and xCdc4β are 99.8% identical (1,623 out of 1,626 nucleotides identical). A single amino acid substitution was made (changing Gly649 to Asp) to the xCdc4α coding sequence used here, as this was conserved in all sequence orthologs. A *X. laevis *ortholog of hCdc4γ was not detected in either stage 7 or stage 20 embryos.

### xCdc4α and xCdc4β proteins are expressed throughout development, including prominent expression in the early neural crest

**Figure 1:**
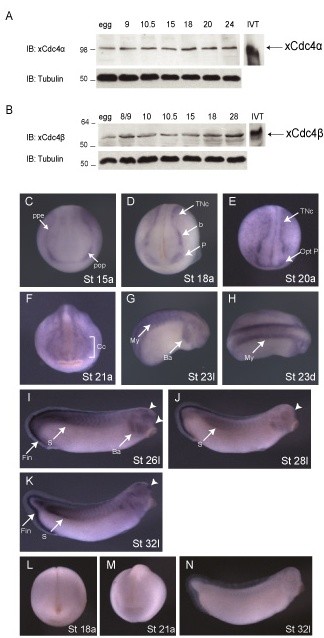
**xCdc4α and xCdc4β protein are expressed throughout development, and transcripts accumulate in the neural crest and neural crest derived tissues**. Expression of **(A) **xCdc4α and **(B) **xCdc4β in staged embryo lysates was determined by immunoblotting (IB) using anti-Cdc4 antibodies. *In vitro *translated (IVT) protein was used as a control. Equal loading was verified by IB for tubulin, with the equivalent of one embryo per lane loaded. **(C-K) **Developmental expression of xCdc4 was determined by whole mount ISH. At stage 15 (C), xCdc4 expression is detected broadly at the preplacodal ectoderm (ppe), in particular in the presumptive optic placode (pop) as well as in the prospective trunk neural crest (TNc). At late neurula stages (D,E), while xCdc4 transcripts continue accumulating in the trunk neural crest and optic placode (Opt P), xCdc4 is also detected in the cranial neural crest, particularly within the branchial aggregates (b). At stage 21 (F), xCdc4 is broadly detected in migrating cranial neural crest (Cc) and placodal regions. At stage 23 (G,H), xCdc4 is expressed in cranial neural crest that populates the branchial arches (Ba) and additionally in the myotome (My). At stage 26 (I), xCdc4 continues to be expressed in the myotome and branchial arches, as well as in several anterior placodes (arrowheads). xCdc4 is additionally expressed in the posterior fin mesenchyme (Fin). From stage 28 to 32 (J,K), xCdc4 expression is downregulated in the branchial arches, while persisting in the anterior placodal region (arrowheads), dorsal somites (s) and dorsal and ventral fin mesenchyme (Fin). Whole mount ISH at the indicated stages using xCdc4β sense probe confirms the specificity of the probe (L-N). Views in (C-N) are indicated bottom right after the stage (St): a, anterior view; d, dorsal view; l, lateral view.

Next, the temporal expression of xCdc4α and xCdc4β was examined during *X. laevis *development. Staged embryo lysates were separated by SDS PAGE and western blotted using antibodies to detect the two different isoforms, xCdc4α and xCdc4β. Anti-Cdc4 3B7 (referred to as anti-Cdc4α) detected both endogenous and *in vitro *translated xCdc4α (Figure 1A). Although unable to detect endogenous levels of xCdc4β, this antibody weakly detected overexpressed xCdc4β (data not shown). Anti-Cdc4 3A9 (referred to as anti-Cdc4β) detected endogenous and *in vitro *translated xCdc4β (Figure 1B) Anti-Cdc4β also detected xCdc4α, although less effectively than anti-Cdc4α (data not shown). Thus, western blotting demonstrates that both xCdc4 isoforms were expressed at all stages tested, from fertilized egg until stage 28.

**Figure 2:**
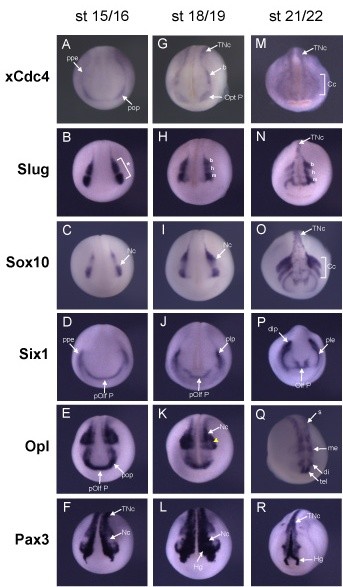
**xCdc4 expression overlaps with expression of markers of neural crest and placodes**. **(A-R) **Comparison of xCdc4 developmental expression (A,G,M) with the expression of neural crest and placodal gene markers in stage 15/16 (B-F), 18/19 (H-L) and 21/22 (N-R) embryos, as labeled. Asterisk indicates premigratory cranial crest; b, branchial crest; Cc, cranial neural crest; di, diencephalons; dlp, dorsolateral placodal area; Hg, hatching gland; h, hyoid crest; m, mandibular crest; me, mesencephalon; Nc, neural crest; Olf P, olfactory placode; Opt P, optic placode; plp, presumptive lens placode; pOlf P, presumptive olfactory placode; ppe, preplacodal ectoderm; pop, presumptive optic placode; s, somites; tel, telencephalon; TNc, trunk neural crest; yellow arrowhead, neural plate border region of the prospective rhombencephalon. Anterior view, dorsal up, stages as indicated.

**Figure 3:**
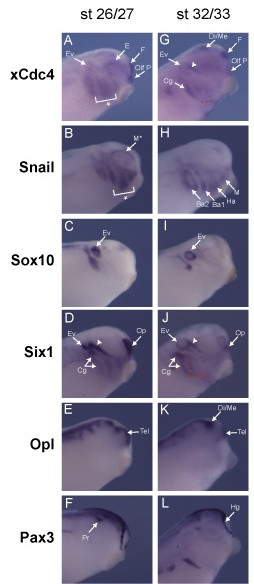
**xCdc4 expression overlaps with expression of neural crest and placodal markers**. **(A-L)**. Comparison of xCdc4 developmental expression (A,G) with the expression of neural crest and placodal gene markers in the mid-tailbud stages 26/27 (A-F) and 32/33 (H-L). Arrowheads indicate the trigeminal placode; white asterisks indicate branchial arches; red asterisks indicate pharyngeal pouches. Ba1-2, branchial arches 1 and 2; Cg, cranial ganglia; Di/Me, diencephalon and midbrain boundary; E, eye; Ev, ear vesicle; F, forebrain; Ha, hyoid arch; Hg, hatching gland; M, mandibular arch; M*, mandibular crest surrounding the eye; Pr, profundal placode; Tel, telencephalon. Lateral view, anterior left, stages as indicated.

The spatial distribution of xCdc4 transcripts during *X. laevis *embryogenesis was also examined by *in situ *hybridization (ISH) using a probe that would recognize both isoforms of xCdc4. Prior to stage 15, xCdc4 transcripts were expressed in a diffuse pattern in the ectoderm (data not shown). At stage 15, while more diffuse background staining remained, transcripts accumulated at the highest level in the neural crest, forming at the preplacodal ectoderm (Figure 1C, ppe) at the border of the epidermis and neural plate and in particular in the presumptive optic placode (Figure 1C, pop). At late neurula stages, xCdc4 transcripts are detected in the trunk (Figure 1D, E, TNc) and branchial neural crest (Figure 1D, b) as well as in the optic placode (Figure 1E, Opt P), and this staining pattern persists until stage 20 (Figure 1D, E). While continuing to be expressed in the neural crest derivatives (cranial neural crest (Cc) and branchial arches (Ba) at later stages (Figure 1F-G)) xCdc4 was additionally expressed in the myotome (Figure 1G, H, My). From stage 26, staining of the posterior somites (s) and the neural crest-derived branchial arches (Ba) persists, while staining in the fin mesenchyme (Fin), another neural crest-derived tissue, becomes prominent (Figure 1I-K). Expression in anterior placodes also becomes pronounced at stage 32 (Figure 1K, arrowheads). Staining was not observed with the sense control probe (Figure 1L-N). In summary, xCdc4 transcripts were detected in the neural crest and placodes, neural crest-derived tissues, and additionally in the brain and somites. We compared the expression of xCdc4 with other markers of neural crest and placodes: *Snail2 *(also known as *Slug*), *Snail*, *Sox10*, *Six1*, *Opl *and *Pax3 *at stages 15/16, 18/19, 21/22, 26/27 and 32/33 [25,30-34] (Figures 2 and 3). At neurula and early tailbud stages, although xCdc4 shows a distinct expression pattern from each of these markers, its expression overlapped both in placodal regions and at the border of the epidermis and neural plate, where early neural crest arises (Figure 2). Similarly, at later stages xCdc4 expression in the branchial arches (Ba), pharyngeal pouches (Pp), cranial ganglia (Cg) and olfactory placode (Olf P) overlapped most significantly with the neural crest marker *Snail *and placodal marker *Six1 *(Figure 3).

**Figure 4:**
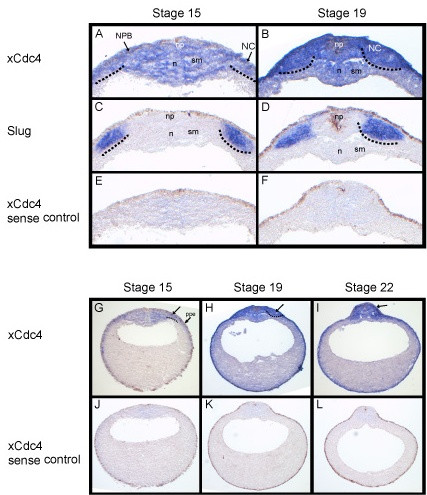
**xCdc4 is found in ectodermal and mesodermal derivatives**. **(A-L) **xCdc4 ISH on transverse sections of stage 15, 18 and 22 embryos (A,B,G-I), including sense control (E,F,J-L) and comparative *Snail2 *expression on stage 15 (C) and stage 18 (D) embryo sections. xCdc4 expression is detected in the neural crest (NC, black arrows), preplacodal ectoderm (ppe) and neural plate border (NPB) as well as in the neural plate (np). In addition to the expression in both superficial and deep layers of the ectoderm, there is also expression of xCdc4 in the notochord (n) and in somitogenic mesoderm (sm).

Surface expression of xCdc4 was clear by whole mount ISH and was most prominent in neural crest and placodal regions. However, we saw less distinct staining more widely across the embryo and in other tissues, such as the myotome and potentially the neural tube (for example, Figure 1G), especially when the staining period was lengthened. Whole mount ISH is most suited to detect expression in tissues near to the embryo surface. To look more closely at xCdc4 expression in deep tissues, we performed ISH on sections. When allowing the ISHs to develop for 2 days, xCdc4 expression was seen throughout dorsal ectodermal and mesodermal tissue of the embryo, including in the neural plate/tube, epidermis, myotome and the notocord, with somewhat weaker staining in the epidermis (Figure 4A, B, G-I), while no staining was seen in the sense control (Figure 4E, F, J-L), demonstrating specificity. In agreement with our whole mount staining (Figure 1D), as the neural tube was closing, expression of xCdc4 appears strongest in areas lateral to the neural plate (Figure 4B) that also express *Snail2 *(Figure 4D), and so are developing neural crest. While other tissues also expressed xCdc4, staining of the neural crest was clear in all embryo sections examined (Figure 4A, B, G-I, arrows).

### xCdc4 degrades Cyclin E and xCdc4ΔFbox mutants act as dominant negatives

Mammalian Cdc4, and in particular Cdc4α, is known to target Cyclin E for ubiquitin-mediated proteolysis [35-38]. We wanted to determine whether xCdc4 could target Cyclin E for degradation in *X. laevis *embryos, both by overexpressing the xCdc4 protein and by knocking out its function. To block xCdc4 function, we initially tried to prevent translation of xCdc4 mRNAs by microinjection of antisense morpholino oligonucleotides. Although xCdc4-directed morpholinos specifically inhibited translation of synthetic mRNA encoding xCdc4α or xCdc4β, demonstrating their functionality, microinjection into embryos had only a small effect on xCdc4α protein levels and no detectable effect on xCdc4β levels, as detected by western blot (data not shown). Morpholinos are only effective when protein levels depend on new translation of mRNAs. Our developmental western blot (Figure 1A, B) indicated that both xCdc4α and xCdc4β are supplied in the egg as maternal stockpiles, and these would not be affected by this antisense strategy.

xCdc4 is a member of the F-box E3 ligase family, and as such, requires an intact F-box to interact with the rest of the SCF complex to target proteins for proteolysis. Deletion of the F-box allows substrate binding but prevents recruitment to the ubiquitination machinery, and this strategy has been successfully used many times to create a dominant negative construct (for example, [9,39]), whose overexpression results in specific substrate hyper-stability. Therefore, we used an F-box deleted dominant negative form of xCdc4 to block the function of endogenous xCdc4 protein.

**Figure 5:**
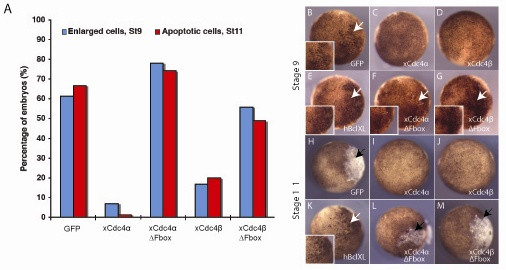
**xCdc4α and xCdc4β block Cyclin E-mediated apoptosis, and this requires an intact F-box domain**. Cyclin E mRNA (1 ng) was co-injected with 1 ng of other mRNAs as indicated into one cell of two-cell-stage embryos. **(A) **Embryos were scored for enlarged cells at stage 9 and apoptotic cells at stage 11 (n = 75-196). **(B-M) **Representative embryos from each injection are shown; enlarged cells are highlighted with white arrows (B,E-G,K) and shown enlarged in insets. Black arrows highlight apoptotic cells (H,L,M).

Cyclin E overexpression in *X. laevis *embryos results in a loss of DNA at early cleavage stages. This leads to a reduction in the rate of cell cleavage, followed by apoptosis of the affected cells at mid-gastrulation [40]. To determine whether xCdc4α and xCdc4β could both act as E3 ligases for Cyclin E *in vivo*, and to demonstrate that xCdc4ΔFbox has lost this activity, we compared their ability to inhibit Cyclin E-mediated apoptosis after micro-injection into *X. laevis *embryos, as compared with the anti-apoptotic protein BclXL. As expected, injection of Cyclin E either alone or with green fluorescent protein (GFP) mRNA resulted in a slowing of blastomere cleavage detectable at stage 9, followed by apoptosis at mid-gastrulation stage 11 (Figure 5B, H). Co-injection of Cyclin E and BclXL resulted in the appearance of larger blastomeres, but these failed to undergo apoptosis later, as expected (Figure 5E, K). Injection of both xCdc4α and xCdc4β along with Cyclin E suppressed the number of embryos with enlarged cells and largely prevented apoptosis (Figure 5A, C, D, I, J), while xCdc4αΔFbox and xCdc4βΔFbox proteins were ineffective at suppressing these phenotypes (Figure 5A, F, G, L, M). Thus, xCdc4α and xCdc4β can act as E3 ligases *in vivo *when overexpressed and require the F-box for this function.

**Figure 6:**
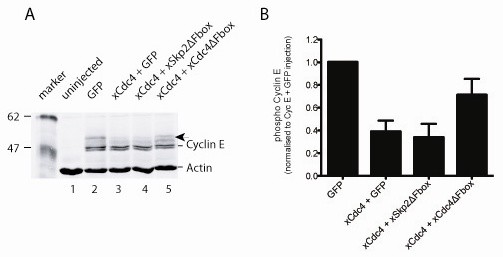
**Phosphorylated Cyclin E protein is degraded by xCdc4**. **(A) **Two-cell-stage embryos were injected with 1 ng of amino-terminally FLAG-tagged Cyclin E mRNA (lanes 2 to 5) and either 1 ng of GFP mRNA (lane 2) or 1 ng of xCdc4 mRNA (lanes 3 to 5). The ability of xCdc4ΔFbox to block Cyclin E degradation was examined by co-injecting 1.5 ng of xCdc4ΔFbox mRNA (lane 5), with 1.5 ng of GFP mRNA or 1.5 ng of Skp2ΔFbox mRNA as controls (lanes 3 and 4). Embryos were allowed to develop to stage 10.5 and immunoblotting was performed for the FLAG epitope, while immunoblotting of actin was performed as a loading control. IR-Dye™-conjugated secondary antibodies were used, enabling accurate quantification of Cyclin E and actin expression. **(B) **The level of phospho-Cyclin E was normalized to the level of actin, then compared to the level of phospho-Cyclin E in GFP-injected embryos, showing mean ± standard error of the mean from four independent experiments.

Cdc4 is thought to target only a phosphoform of Cyclin E for destruction [35-38]. To look directly at the effect of xCdc4 on degradation of Cyclin E protein by western blot, 1 ng of RNA encoding amino-terminal FLAG Cyclin E was injected into fertilized eggs along with mRNAs encoding xCdc4 (using the α isoform) or GFP, as an injection control (Figure 6), and embryos were allowed to develop to stage 10.5. Embryo lysates were prepared and immunoblotting was performed to detect the FLAG epitope on Cyclin E.

Cyclin E migrated as multiple bands in GFP-injected embryos (Figure 6A, lane 2), and these are likely to be phospho-forms of the protein [41]. When co-injected with Cyclin E mRNA, xCdc4 expression resulted in a reduction in the abundance of predominantly the slower-migrating phospho-forms of Cyclin E protein (Figure 6A, lane 3). Densitometry analysis, normalizing to actin expression in the same samples, confirmed that phosphorylated forms of Cyclin E are degraded by xCdc4. This is in agreement with previous results, which demonstrated a requirement for Cyclin E phosphorylation to direct Cdc4-mediated degradation [9,38,42]. These results verify that xCdc4 is functionally orthologous to mammalian Cdc4 in its ability to degrade phospho-forms of Cyclin E.

To confirm that the F-box deletion mutant of xCdc4 (xCdc4ΔFbox) possessed dominant negative activity, its ability to inhibit Cyclin E degradation mediated by wild-type xCdc4 was assessed. Cyclin E mRNA was co-injected with xCdc4 along with one of the following: xCdc4ΔFbox, xSkp2ΔFbox, an F-box mutant of the related SCF E3 ligase Skp2, or GFP (Figure 6A, lanes 2 to 5). As expected, overexpression of either xSkp2ΔFbox or GFP had no effect on phospho-Cyclin E degradation by xCdc4. In contrast, degradation of Cyclin E by xCdc4 was inhibited by co-injection of xCdc4ΔFbox (lane 5), confirming that this mutant acts as a dominant negative form of xCdc4 in the embryos. When phospho-Cyclin E levels were normalized to the levels in embryos injected with Cyclin E and GFP, xCdc4 and GFP co-injection led to a 60% reduction in Cyclin E levels (Figure 6B). When xCdc4 was co-injected with xSkp2ΔFbox, a similar reduction in Cyclin E levels was observed (70%). However, coexpression of xCdc4ΔFbox with xCdc4 partially rescued the degradation of Cyclin E. On average, there was a 30% reduction in Cyclin E levels compared to Cyclin E- and GFP-injected embryos. In addition, we also found that xCdc4 targeted endogenous Cyclin E for degradation (data not shown), again predominantly reducing the slower-migrating phospho-form of the protein.

At these early developmental stages, Cyclin E is expressed throughout the ectoderm [43], so may act as a target for xCdc4. Since Cdc4 is known to regulate the stability of other proteins that regulate cell proliferation (for a review, see [10]), we investigated whether xCdc4 modulated cell cycle progression in the early embryo. As xCdc4α and xCdc4β share the same substrate binding regions, xCdc4βΔFbox (hereafter known as xCdc4ΔFbox) would be expected to inhibit the activity of both xCdc4 isoforms, and was used throughout in this study. Indeed, xCdc4αΔFbox gave similar results to xCdc4βΔFbox (data not shown).

### xCdc4ΔFbox does not affect cell cycle in the embryonic ectoderm

**Figure 7:**
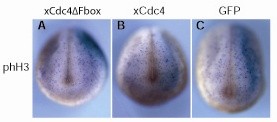
**xCdc4ΔFbox does not affect the cell cycle**. xCdc4ΔFbox or xCdc4 mRNA (1 ng) was injected into one cell of two-cell-stage embryos. GFP mRNA (1 ng) was injected as a control, and β-gal mRNA was injected as a lineage tracer (light blue unilateral staining). Whole mount antibody staining against phH3 was performed to detect mitotic cells. The average percentage difference in the number of mitotic cells on the injected side, compared to the uninjected side, was calculated as indicated in Materials and methods. Representative embryos are shown (anterior view, dorsal up, injected side right).

To investigate the effect of xCdc4 activity on cell proliferation, mRNA encoding xCdc4β or xCdc4ΔFbox was injected unilaterally into two-cell-stage embryos with GFP mRNA injected as a control. Whole mount staining using an antibody against phosphorylated histone H3 (phH3) was used to examine the number of mitotic cells [44]. No change in the number of mitotic cells in the neural folds was observed following overexpression of xCdc4ΔFbox or xCdc4 (Figure 7). To quantify this, the number of mitotic cells in the neural folds was counted on the injected and uninjected sides, and the percentage difference was calculated for each embryo. The average percentage change for xCdc4ΔFbox (-2.8 ± 3.9%) and xCdc4 (-9 ± 1.2%) overexpression was not significantly different from that found after GFP overexpression (-8.3 ± 1.5%, n = 84 to 91). Therefore, overexpression of xCdc4ΔFbox did not perturb the cell cycle. Indeed, we saw no difference in the size of the blastomeres on the injected versus the uninjected side of the embryo (data not shown and Figure 7), confirming this conclusion.

### xCdc4ΔFbox inhibits neural crest development

**Figure 8:**
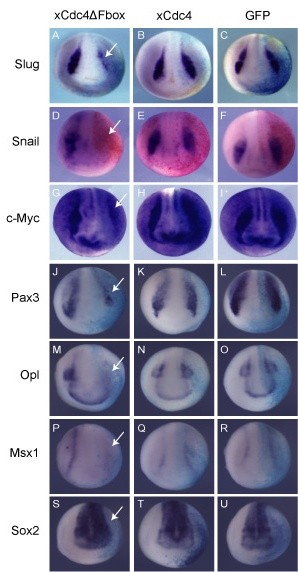
**xCdc4ΔFbox inhibits neural crest development**. xCdc4ΔFbox or xCdc4 mRNA (1 ng) was injected into one cell of two-cell-stage embryos. GFP mRNA (1 ng) was injected as a control, and β-gal mRNA was injected as a lineage tracer (light blue or red unilateral staining). **(A-U) **Whole mount ISH was performed for *Snail2 *(A-C), *Snail *(D-F), *c-Myc *(G-I), *Pax3 *(J-L), *Opl *(M-O) and *Msx1 *(P-R), and the early neuronal marker *Sox2 *(S-U). Representative embryos are shown (anterior view, dorsal up, injected side right).

xCdc4 is expressed prominently in the neural crest throughout early development (Figures 1 and 2). In order to examine the effect of inhibition of xCdc4 on neural crest development, xCdc4ΔFbox was overexpressed in developing embryos and compared to xCdc4 and GFP injections as controls. After injection of mRNAs into one cell of two-cell embryos, and subsequent development to neural plate stage 15, whole mount ISH for the neural crest markers *Snail2*, *Snail *and *c-Myc *were performed. Overexpression of xCdc4ΔFbox inhibited formation of the neural crest as measured by decreased *Snail2 *and *Snail *expression (Figure 8A, D), while wild-type xCdc4 and GFP had very little effect (Figure 8B, C, E, F). In addition, *c-Myc*, a very early marker of neural crest [28], was also reduced by xCdc4ΔFbox (Figure 8G), but not by xCdc4 or GFP (Figure 8H, I).

To quantify the reduction in expression of neural crest markers brought about by overexpression of xCdc4ΔFbox, the area of staining of *Snail2*, *Snail *and *c-Myc *on the injected side of each embryo was measured and expressed as a ratio of the area on the uninjected side. A ratio less than 1 indicated a reduction in staining on the injected side compared to the uninjected side. The mean ratio was then calculated for at least two independent experiments, and compared with the ratio for GFP-injected embryos (which had minimal effect).

Overexpression of xCdc4ΔFbox blocked neural crest development, as determined by expression of *Snail2*, *Snail *and *c-Myc*, resulting in an average 57% reduction in *Snail2 *staining on the injected side of the embryo, compared with embryos injected with GFP alone. Overexpression of xCdc4 had essentially no effect on *Snail2 *staining (n = 58 to 122). Similarly, overexpression of xCdc4ΔFbox reduced *Snail *and *c-Myc *staining on the injected side of the embryo by 80% and 67% respectively, when compared to embryos injected with GFP alone (n = 30 to 52 for both).

**Figure 9:**
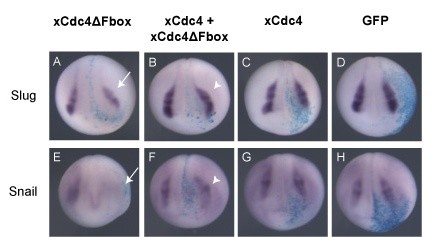
**Loss of neural crest induced by xCdc4ΔFbox is rescued by co-injection of xCdc4**. xCdc4ΔFbox mRNA (1 ng) was co-injected with either 1 ng of xCdc4 or 1 ng of GFP mRNA into one cell of two-cell-stage embryos. As controls, 1 ng of xCdc4 mRNA was co-injected with 1 ng of GFP mRNA, as well as injection of 2 ng of GFP mRNA as a separate control. β-gal mRNA was injected as a lineage tracer. **(A-H) **Embryos were allowed to develop to stages 18/19 and whole mount ISH was performed for the neural crest markers *Snail2 *(A-D) and *Snail *(E-H). Representative embryos are shown (anterior view, dorsal up, injected side right). Arrows indicate loss of expression of each marker on the injected side of xCdc4ΔFbox expressing embryos. The reduction in expression of each marker on the injected side of xCdc4ΔFbox expressing embryos is rescued by co-injection of xCdc4 mRNA (arrowheads).

To confirm that xCdc4ΔFbox was acting as a dominant negative, we determined whether co-injection with wild-type xCdc4 could rescue the reduction in neural crest. In this experiment, xCdc4ΔFbox significantly reduced *Snail2 *and *Snail *expression in 34% and 67% of embryos, respectively, compared to only 12% and 6%, respectively, when the wild-type xCdc4 was co-injected with xCdc4ΔFbox (n = 18 to 21; Figure 9). This rescue demonstrates that xCdc4ΔFbox can act as a dominant negative in the presence of wild-type xCdc4. Indeed, as endogenous xCdc4 is likely to be expressed at a much lower concentration than that expressed from micro-injected mRNA, xCdc4ΔFbox is likely to be much more effective at inhibiting the endogenous xCdc4 protein.

As our sections revealed rather broad expression of xCdc4 in ectodermal and mesodermal derivatives, we investigated whether xCdc4ΔFbox affected patterning and/or specification of other tissues. We saw that anterior-posterior patterning and mesoderm development of the embryos were unaffected by overexpression of either wild-type xCdc4 or xCdc4ΔFbox, as determined by *Otx2*, *Engrailed2*, *Krox20*, *MyoD*, *Heavy chain myosin *(*HCM*) and *epidermal keratin *expression (Additional files 2 and 3). Moreover, *Sox2*, a marker of neural plate neuronal precursors, and *Neural beta-tubulin *(*NβT*), a marker of neuronal differentiation, were also unaffected (Figure 8S; Additional file 3). This suggested that the effect of xCdc4ΔFbox overexpression on neural crest development was not dependent on either early patterning events or secondary effects resulting from induction of mesoderm, and was indeed specific to the neural crest. This effect is also specific to xCdc4ΔFbox; the Skp2ΔFbox mutant had no effect on *Snail2 *expression (Additional file 4).

We investigated whether xCdc4 activity was required for expression of other regulators of neural crest formation lying upstream of *Snail2 *and *Snail *(Figure 8). At stage 15, *Pax3 *(53%) and *Msx1 *(100%) showed significant reduction in the presence of xCdc4ΔFbox but not wild-type xCdc4, indicating that xCdc4 plays a broad role in controlling expression of regulators of neural crest. Interestingly, xCdc4ΔFbox inhibited neural crest expression of *Opl *in some embryos (62%), but did not have a significant effect on its placodal expression (Figure 8M). This is supported by the failure of xCdc4ΔFbox to affect the expression of the placodal marker *Six1 *(Additional file 5). Thus, xCdc4 does not play an essential role in specification of all the tissues in which it is expressed, but does have an essential role in formation of the neural crest.

**Figure 10:**
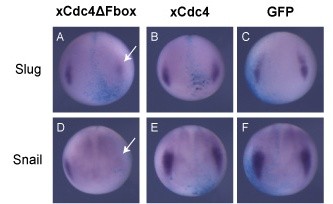
**xCdc4ΔFbox inhibits neural crest specification**. xCdc4ΔFbox or xCdc4 mRNA (1 ng) was injected into one cell of two-cell-stage embryos. GFP mRNA (1 ng) was injected as a control, and β-gal mRNA was injected as a lineage tracer (light blue unilateral staining). **(A-F) **Embryos were allowed to develop to stage 13/14 and the expression of *Snail2 *(A-C) and *Snail *(D-F) in the prospective neural crest region was examined by whole mount ISH. Representative embryos are shown (anterior view, dorsal up, injected side right). Arrows indicate loss of expression of each marker on the injected side of xCdc4ΔFbox expressing embryos.

xCdc4 is expressed broadly and diffusely before stage 15, and while expression is seen in other tissues, transcripts concentrate in the placodes and neural crest derivatives as the neural tube closes (Figures 1, 2, and 4). The first signs of neural crest specification occur earlier, so we investigated whether xCdc4 was required for establishment or maintenance of neural crest identity. To this end, we investigated the effect of xCdc4ΔFbox expression on earlier marker expression in the prospective neural crest (Figure 10). After overexpression of xCdc4ΔFbox, we saw a reduction in *Snail2 *(44%; n = 32) and *Snail *(58%; n = 26) expression by stage 13/14 that was not seen with the wild-type protein (both 100% no significant reduction; n = 25 and n = 35, respectively), indicating that xCdc4 has a role early in neural crest specification, and is not solely required for its maintenance.

**Figure 11:**
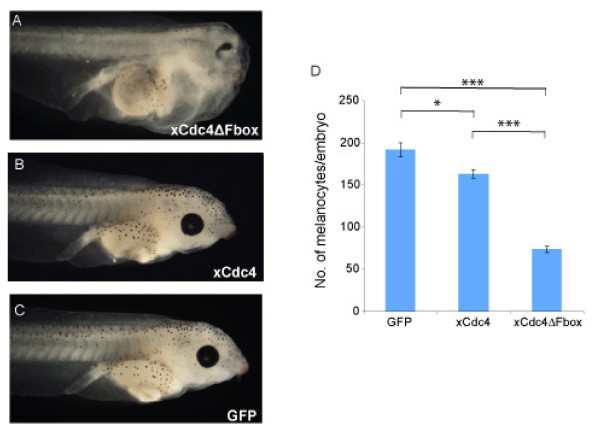
**xCdc4ΔFbox impairs melanocyte development**. xCdc4ΔFbox, xCdc4 or control GFP mRNA (0.5 ng) were injected into each of the two dorsal cells of four-cell-stage embryos. (A-C)Embryos were allowed to develop to stage 43 and subsequently the distribution and number of melanocytes were compared: xCdc4ΔFbox (A), xCdc4 (B) and GFP (C). (A-C) Representative embryos are shown (lateral views, anterior side right). (D)The average number of melanocytes for each condition was calculated and the mean ± standard error of the mean from two independent experiments are shown (n = 31 to 57). There was a significant decrease in the number of melanocytes following injection with xCdc4ΔFbox when compared to GFP or xCdc4 (***P << 0.0001), and a smaller decrease following injection with xCdc4 when compared to injection with GFP (*P < 0.01).

To determine the effect of inhibiting xCdc4 on later neural crest derivatives, we allowed embryos injected in both dorsal cells of four-cell-stage embryos to develop until stage 43, and then investigated the differentiation of melanocytes and formation of cartilage, both neural crest-derived tissues. We saw that embryos injected with GFP had a normal number and distribution of melanocytes. In contrast, after injection of xCdc4ΔFbox, embryos showed significant reduction and an abnormal distribution of melanocytes (Figure 11; n = 31 to 57, *P *<< 0.0001)) compared with the GFP-injected controls. A much smaller decrease in the number of melanocytes was also observed following injection with xCdc4 when compared with the GFP control (*P *< 0.01). As xCdc4ΔFbox-injected embryos looked abnormal, with a significant reduction of head morphology, we examined the effect of xCdc4ΔFbox on cartilage formation by staining the embryos with alcian blue. We found that most xCdc4ΔFbox-injected embryos showed compacted head cartilage with weaker cartilage staining compared to xCdc4- or GFP-injected embryos (data not shown).

We also noted that embryos that were expressing xCdc4ΔFbox show other developmental abnormalities, including oedema and a high frequency of reduced eyes (Figure 11A). These phenotypes, which we have not been characterized further, may be secondary to loss of neural crest or may result from a later requirement of xCdc4 activity in other tissues where it is expressed, for example, the eye field (Figure 1I). As injected mRNA is not thought to persist much beyond tailbud stages, these defects most likely result from a requirement for xCdc4 at these earlier stages.

**Figure 12:**
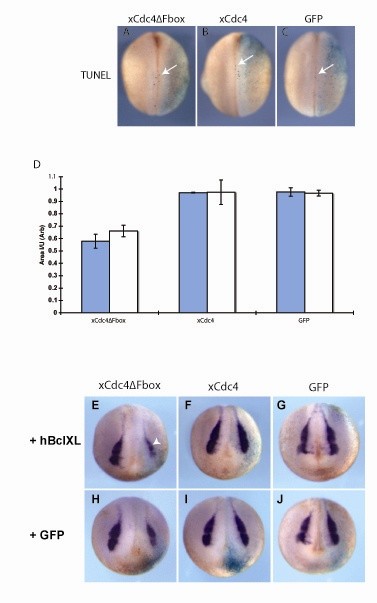
**xCdc4ΔFbox does not reduce neural crest by inducing apoptosis**. **(A,B) **xCdc4ΔFbox or xCdc4 mRNA (1 ng) was injected into one cell of two-cell-stage embryos. **(C) **GFP mRNA (1 ng) was injected as a control, and β-gal mRNA was injected as a lineage tracer (light blue unilateral staining). Apoptosis was assessed using TUNEL staining. Arrow indicates TUNEL-positive cells. Representative embryos are shown (anterior view, dorsal up, injected side right). **(D) **The area of *Snail2 *staining on the injected side was expressed as a ratio of the area on the uninjected side, and for each experiment an average ratio was obtained (see Materials and methods). Mean ± standard error of the mean ratio from two independent experiments are shown (n = 57-82). **(E-J) **xCdc4ΔFbox, xCdc4 or GFP mRNA (1 ng) was injected into one cell of two-cell-stage embryos, along with 1 ng of hBclXL or 1 ng of GFP mRNA as indicated, with β-gal mRNA as a lineage tracer (light blue unilateral staining). Whole mount ISH for *Snail2 *was performed on stage 18 embryos. The reduction in expression of *Snail2 *on the injected side of xCdc4ΔFbox expressing embryos is not rescued by co-injection of hBclXL (arrowheads). Representative embryos from the indicated injections are shown (anterior view, dorsal up, injected side right).

Since xCdc4ΔFbox did not result in an increase in cycling cells that could disrupt neural crest differentiation, we investigated the possibility that xCdc4ΔFbox results in apoptosis of cells destined to become neural crest. TUNEL (terminal deoxynucleotidyltransferase-mediated dUTP-biotin nick end labeling) staining was used to detect apoptotic cells following mRNA injection. No difference in TUNEL staining was observed following overexpression of xCdc4ΔFbox (Figure 12A-D). Approximately 70% of embryos had TUNEL positive cells (n = 100 to 110), but in no case were differences in TUNEL staining observed, either in the neural folds or in the embryo as a whole.

We were surprised at the low frequency of TUNEL-positive cells detected in this assay, although embryos that had been wounded were used as a positive control and were highly TUNEL-positive (data not shown). Therefore, an alternative assay for apoptosis was also used. We took advantage of the fact that hBclXL can block apoptosis in *X. laevis *[19,40]. We checked whether *Snail2 *expression in xCdc4ΔFbox injected embryos could be rescued by co-expression of hBclXL. xCdc4ΔFbox, xCdc4 or GFP (1 ng) were injected unilaterally into two-cell-stage embryos. In addition, 1 ng of hBclXL or GFP were co-injected as appropriate. Whole mount ISH for *Snail2 *was performed on stage 18 embryos, and *Snail2 *staining in xCdc4ΔFbox/hBclXL-injected embryos was compared to xCdc4ΔFbox/GFP-injected embryos (n = 57 to 82; Figure 12E-J). In these experiments, xCdc4ΔFbox/GFP-injected embryos displayed an average 32% reduction in *Snail2 *staining, compared to GFP-injected embryos (mean ± SEM ratios were 0.66 ± 0.05 and 0.97 ± 0.02, respectively). However, no rescue of *Snail2 *staining was seen when hBclXL was co-injected with xCdc4ΔFbox, with an average 41% reduction in *Snail2 *staining on the injected side compared to embryos injected with GFP/hBclXL. Thus, loss of neural crest after inhibition of xCdc4 activity is not due to tissue loss by apoptosis.

In summary, we have identified two homologues of Cdc4 in *X. laevis *that show dynamic expression in the early embryo, in particular in the developing neural crest and its derivatives. While unexpectedly having no effect on cell cycling, blocking xCdc4 function by overexpression of a dominant negative form of the protein resulted in inhibition of specification and formation of the neural crest. This had long-term consequences for the formation of neural crest derivatives such as melanocytes and cartilage. Thus, regulated and specific proteolysis by xCdc4 plays an essential function in early development distinct from its well-established role in regulating cell division.

## Discussion

This work has identified a novel role for the F-box protein xCdc4 in neural crest development in *X. laevis*. Cdc4 orthologs in vertebrates have attracted considerable interest due to the plethora of substrates they degrade, and the fact that Cdc4 is a haplo-insufficient tumor suppressor protein [10,16]. However, the embryonic lethality of Cdc4 knock-out mice precluded a detailed analysis of the role of this protein in development [17].

*X. laevis *Cdc4 is likely to be orthologous to vertebrate Cdc4 for several reasons. Firstly, xCdc4 is highly conserved compared to human Cdc4, both in terms of sequence and apparent gene organization. Secondly, xCdc4α and xCdc4β are capable of degrading Cyclin E, a known substrate of mammalian Cdc4. This validates the use of *X. laevis *to study the developmental function of Cdc4. In contrast, recovered *Cdc4*^-/- ^mouse embryos show profound vascular defects [17], precluding study in other developmental processes. Developing amphibian embryos do not require a functional vasculature until later embryonic stages, making this an excellent system for studying other functions of Cdc4.

In this work, we report the isolation of two xCdc4 isoforms, xCdc4α and xCdc4β. The genes encoding them are almost identical from the region corresponding to the second exon (1,622 out of 1,625 nucleotides identical). This strongly suggested that the structure of the *Xenopus *gene was conserved compared to mammals. In humans and mice, the Cdc4 locus encodes three isoforms of Cdc4; designated α, β and γ. These are produced by alternative splicing of three unique 5' exons to ten common 3' exons, yielding proteins that differ only at their amino termini. Although our data suggest that this gene structure is preserved, no xCdc4γ isoform was detected during this work. In addition, *in silico *analysis, using the Ensembl genome browser, failed to detect sequences corresponding to the γ specific exon.

To check if xCdc4 was functionally orthologous to vertebrate Cdc4, the ability of xCdc4 to degrade Cyclin E was examined. xCdc4 overexpression reduced the abundance of Cyclin E, compared to embryos co-injected with GFP, preferentially reducing the abundance of the slower migrating hyper-phosphorylated form of Cyclin E (Figure 6A). This is in agreement with previous findings that Cdc4 only interacts with phosphorylated Cyclin E [35-38]. xCdc4 also degrades Cyclin E *in vivo*, while xCdc4ΔFbox does not (Figure 6).

The spatio-temporal expression of xCdc4 was examined by immunoblotting for xCdc4 isoforms, and by whole mount ISH (Figure 1). Using antibodies that detected xCdc4, both isoforms were expressed at all stages tested. xCdc4α migrated at an apparent molecular weight of 100 kDa, despite having a predicted molecular weight of 79 kDa (Figure 1A). This is in keeping with a previous report that showed that human Cdc4α migrated with an apparent molecular weight of 110 kDa, although its predicted molecular weight was 80 kDa [9]. In contrast, xCdc4β consistently migrated slightly faster than its predicted molecular weight of 62 kDa (Figure 1B).

Although expressed across the embryo, the neural crest and placodes were the first sites where xCdc4 transcripts accumulated most prominently (Figures 1, 2 and 4). Subsequently, transcripts were detected in the branchial arches and fin mesenchyme, which are both neural crest derived tissues, as well as in the somites and brain, and co-localized in many areas with other known markers of neural crest and placodes (Figures 1 and 2). In order to examine the function of xCdc4 during development, gain and loss of function experiments were performed. Translation-blocking morpholinos did not inhibit expression of endogenous xCdc4α and xCdc4β (data not shown). One possible explanation for this was the maternal expression of the proteins. Therefore, an F-box deletion mutant was used as an alternative way to inhibit the function of xCdc4. xCdc4ΔFbox acted as a dominant negative inhibitor of xCdc4's ability to degrade Cyclin E (Figure 6) because it retains its ability to bind to substrate but cannot recruit the substrate to the rest of the SCF complex. The specificity of F-box proteins resides in their substrate binding site, and F-box deletion mutants, which bind specifically to their substrate but not to the Skp1 component of the SCF E3 ligase complex, have been widely employed as reagents to block degradation of specific F-box targets. Confirming this, xSkp2ΔFbox did not inhibit xCdc4-mediated degradation of the protein. As xCdc4 is expressed strongly in the neural crest, we investigated the effect of overexpression of xCdc4ΔFbox on development of this tissue.

Inhibition of xCdc4, using xCdc4ΔFbox, inhibited neural crest development, as determined by *Snail2 *and *Snail *ISHs (Figures 8, 9 and 10). This inhibition of neural crest development occurred at an early stage, upstream of *c-Myc*, reducing expression of a number of proteins acting as regulators of *Snail2*, *Snail *and *c-Myc *(Figure 8). This indicates that xCdc4 is required for establishment of neural crest identity, rather than simply maintenance of that identity. Although expressed more broadly in ectodermal and mesodermal derivatives, we did not detect a requirement for xCdc4 function in specification or differentiation of other tissues, nor in patterning of the neural tube (Additional files 2, 3 and 5), strongly indicating a tissue-autonomous role in the neural crest.

The most well characterized role of Cdc4 is in regulating the cell cycle, and we investigated whether xCdc4 regulates cell cycling in the early *X. laevis *embryo. xCdc4ΔFbox overexpression did not perturb the cell cycle as determined by phH3 staining in these early embryos (Figure 7). The results from phH3 staining are consistent with previous observations that cell proliferation has a minor role in neural crest development; neural and neural crest induction has been reported to proceed normally when cell division was blocked from mid-gastrula stages onwards [19,45].

xCdc4ΔFbox does not inhibit neural crest development through activation of an apoptotic program. The evidence for this is threefold: xCdc4ΔFbox overexpression did not lead to a loss of β-gal staining expressed from co-injected mRNA; there was no change in TUNEL staining (Figure 12A-D); and finally, co-injection of hBclXL did not affect the ability of xCdc4ΔFbox to block neural crest development (Figure 12E-J). *X. laevis *embryos can undergo apoptotic cell death from stage 10.5 onwards [46-48]. TUNEL analysis during embryonic development showed that only 50 to 60% of embryos had TUNEL-positive cells in the ectoderm prior to stage 18 [49]. After this stage a higher percentage of embryos were TUNEL-positive, but individual embryos displayed less TUNEL staining. In terms of neural crest development, Sox10 and Id3 depletion have been reported to increase TUNEL staining in the neural crest, but this was coupled to a reduction in cell proliferation [50,51]. However, it has been reported that inhibition of the cell cycle reduces clearance of apoptotic cells, meaning that cell cycle inhibition in these embryos may have led to increased TUNEL staining [52].

Previous studies have reported increased levels of apoptosis occurring in neural folds rather than other areas of the embryonic ectoderm [47,49,53], and a number of genes involved in neural crest development possess anti-apoptotic activity (for example, [53,54]). It is possible that apoptosis regulates neural crest development in a stage-specific manner. For example, it may be important in defining the neural crest boundary, rather than for neural crest induction. However, as xCdc4 acts at an early stage of neural crest development, regulation of apoptosis may not be involved.

The identification of Cdc4 as a regulator of neural crest development adds to existing evidence that the ubiquitin proteasome system plays a role in the development of this tissue. Overexpression of dominant negative Cullin1, which is predicted to inhibit all SCF E3 ligases, expanded the neural crest in *X. laevis*. This was primarily due to stabilization of the Wnt pathway component β-catenin, although other substrates are likely to have been involved [55]. Recently, the F-box protein Ppa was shown to regulate neural crest development by degradation of Snail2 in *X. laevis*. Overexpression of Ppa inhibited neural crest development [26]. The related protein Snail is degraded by the F-box protein β-TRCP in tissue culture cells [56]. Thus, a number of proteins involved in neural crest development display dynamic expression patterns, and regulation by ubiquitin-mediated proteolysis is emerging as a method to achieve this through activity of different E3 ligases that target distinct substrates.

What substrates is Cdc4 targeting to influence neural crest formation? c-Myc regulates neural crest development [19,57] and can be degraded by Cdc4 [11,29], but we saw that xCdc4ΔFbox decreased c-Myc mRNA (Figure 8), indicating that xCdc4 also acts upstream of c-Myc expression. Indeed, xCdc4 acts upstream of early regulators of neural crest, such as Pax3 and Msx1 (Figure 8). We hypothesized that xCdc4 was degrading a negative regulator of neural crest development. c-Jun is another known target of Cdc4 [12] and is expressed at the right time and place to be regulating neural crest formation, although its exact role is poorly understood [58,59]. We saw that overexpression of c-Jun did indeed inhibit *Snail2 *expression (Additional file 6). However, when we tested whether xCdc4 could target c-Jun protein for degradation in embryos by co-injection of mRNAs encoding these proteins, and performing immunoblotting against HA-tagged c-Jun, we saw no effect of xCdc4 on c-Jun levels (data not shown). From these results, it is not clear whether c-Jun is a target for Cdc4 in this developmental context, and in any case, an essential role for c-Jun in regulating neural crest formation in *X. laevis *has not been clearly demonstrated.

Identification of E3 ligase targets is challenging, and co-factors required for protein degradation may be active only in certain contexts. For example, it has been noted that Cdc4-mediated degradation of c-Jun in tissue culture cells required co-expression of Glycogen synthase kinase 3β (GSK3β) [60]. It will be important to now identify the *in vivo *targets of Cdc4 that regulate formation of the neural crest if we are to have a fuller understanding of the role of selective protein degradation in the development of this tissue.

## Conclusions

Here we identify xCdc4 as a novel regulator of neural crest development in *X. laevis*, acting early in neural crest formation, potentially by regulation of c-Jun. These results demonstrate that Cdc4's role as a tumor suppressor protein may extend beyond its ability to regulate the cell cycle to an ability to directly regulate tissue differentiation.

## Materials and methods

### Plasmids and constructs

Expression of constructs was verified by immunoblotting embryo lysates after microinjection of capped mRNA.

*X. laevis Cdc4β *(*xCdc4β*) was cloned by PCR from stage 20 cDNA. Primers used to obtain the full length coding sequence were: forward, 5'-ATGGGCTTCTACGGCAC-3'; reverse, 5'-CCTTCACTTCATGTCCACGTC-3'. *xCdc4β *was cloned into pCS2+repaired to synthesize capped mRNA for microinjection. An F-box deletion mutant of *xCdc4β *(*xCdc4βΔFbox*) was produced by PCR and triple ligation strategy. The F-box region of *xCdc4β *(nucleotides 379 to 516 inclusive) was replaced with an *Ase*I site (ATTAAT, amino acids Ile and Asn).

Primers 5'-GCAACCGAATTCACCACCATGGGCTTCTACGGCAC-3' and 5'-GGTTGCATTAATAAAGTCCCGCTGAAACTGG-3' were used to amplify *xCdc4β *upstream of the F-box, and 5'-GCAACCATTAATGAAGATGGGATCGATGAGC-3' and 5'-GGTTGCCTCGAGTCACTTCATGTCCACGTC-3' were used to amplify *xCdc4β *downstream of the F-box.

*X. laevis Cdc4α *(*xCdc4α*) was similarly cloned using cDNA derived from stage 7 embryos. Primers used to obtain the full length coding sequence were: forward, 5'-GCTGGCTTTTGGAAATGAATCAGG-3'; reverse, 5'-CTTCACTTCATGTCCACATCAAAGTCC-3'. *xCdc4α *was cloned into pGEM-T Easy (Promega, Madison, WI, USA) downstream of the SP6 promoter. The GenBank accession number is DQ666345. A single point mutation was introduced into this sequence (nucleotide G1946A in the coding sequence, resulting in G649D in the protein), to introduce an Asp residue that is conserved amongst vertebrates, to correct what was likely a cloning error; xCdc4α (G) was much less potent at inhibiting formation of neural crest compared to xCdc4α (D) (data not shown). XCdc4α (D) also degraded Cyclin E more efficiently than xCdc4α (G) (data not shown). Site directed mutagenesis was performed using a QuikChange^® ^Multi Site Directed Mutagenesis kit (Stratagene, La Jolla, California) according to the manufacturer's instructions. *xCdc4αΔFbox *was produced by an identical method to that described for *xCdc4βΔFbox*. *X. laevis Skp2ΔFbox *has been described elsewhere [39].

*X. laevis *c-Jun in pCS2+ (GenBank accession number AJ243954) was a kind gift of Professor Walter Knochel (Institute of Biochemistry, University of Ulm, Germany).

### Antibodies

Anti-Cdc4 monoclonal antibodies (3B7 and 3A9) were a kind gift from Dr Axel Behrens (CRUK). Anti-FLAG M2 conjugated to horse radish peroxidase (A8592; used at 1:1,000), anti-tubulin B512 (T5168; used at 1:2,000) and anti-rabbit IgG conjugated to alkaline phosphatase (A9919; used at 1:1,000) were from Sigma (St Louis, MO, USA). Horse radish peroxidase conjugated anti-rabbit and mouse IgG (NA943 and NA931; used at 1:5,000) were from Amersham (GE Healthcare, Uppsala, Sweden), rabbit anti-phH3 (06570; used at 1:1,000) was from Upstate (Millipore, Billerica, MA, USA) and alkaline phosphatase conjugated anti-digoxigenin fab fragments (11093274910; used at 1:5,000) were from Roche (Basel, Switzerland).

### *Xenopus laevis *embryo manipulation

*X. laevis *embryos were obtained by hormone-induced egg laying and *in vitro *fertilization by standard methods. Unilateral injections into the animal pole of two-cell-stage embryos (unless otherwise indicated) were carried out with *in vitro *transcribed capped mRNA (Ambion, Austin, TX, USA). mRNA was injected at doses of up to 2 ng in a volume of 10 nl, and 0.5 ng of β-gal mRNA was co-injected as a lineage tracer. GFP mRNA was injected as a control. Embryos were staged according to [61] and grown to the required stage. They were fixed in MEMFA (4% formaldehyde, 100 mM MOPS, 2 mM EGTA, 1 mM MgSO_4_, pH 7.4), washed in phosphate-buffered saline (PBS)/2 mM MgCl_2_, and stained with 1 mg/ml X-gal (5-bromo-4-chloro-3-indolyl-beta-D-galactopyranoside) in X-gal mixer (5.35 mM K_3_Fe(CN)_6_, 5.35 mM K_4_Fe(CN)_6_, 1.2 mM MgCl_2_, 0.1% sodium deoxycholate, 0.2% NP-40 in PBS). Alternatively, embryos were stained with 1.7 mg/ml Red-gal^® ^(6-chloro-3-indolyl-beta-D-galactopyranoside) in X-gal mixer. Embryos were washed in PBS, dehydrated in methanol and stored at -20°C.

### *In situ *hybridization

Whole mount ISH was performed using a BioLane™ HTI *in situ *robot (Holle and Huttner (Tubingen, Germany). The washes and composition of solutions were as described in [62], with some modifications in the protocol. The RNase step was omitted, and embryos were blocked with 2% Blocking Reagent (Roche) and 20% heat inactivated lamb serum in maleic acid buffer. Incubation with 1:5,000 anti-digoxigenin was performed in the same solution. The color reaction was terminated using PBS washes and embryos were re-fixed in MEMFA. Embryos were bleached as described in [62]. *xCdc4 *in pBSK+ was linearized with *Pst*I and transcribed T3 for antisense, and linearized with *Not*I and transcribed T7 for sense. The following probes were used: *c-Myc*, *MsxI *[53], *Opl*/*Zic1 *[32], *Pax3 *[30], *Snail*, *Six1 *[34], *Sox2*, *Sox10 *[31] and *Snail2 *[25].

To quantify the area of *Snail2 *expression, Openlab™ software (Improvision/Perkin Elmer, Waltham, MA, USA) was used to select the area of *Snail2 *staining on the injected (I) and uninjected (U) side, using all injected embryos unless they were damaged. For each experiment the average ratio of I/U was determined for each injection to determine the change in *Snail2 *staining on the injected side, a ratio of <1 indicating *Snail2 *reduction. For at least two experiments, a mean ratio ± standard error of the mean (SEM) for each injection was calculated by taking the mean of the average ratios, and a Student's *t*-test performed. The ratios were compared to GFP-injected control embryos.

ISH on sectioned embryos was performed as described in [63]. Stage 15 to 22 embryos were fixed in MEMFA, embedded in a paraffin and beeswax solution and sectioned using a Leica microtome. The ISH was performed on 14-μm sections using *xCdc4 *and *Snail2 *probes. The slides were mounted using Aquamount (BDH-Merck, VWR International, West Chester, PA, USA).

### Whole mount antibody staining

Whole mount antibody staining, using anti-phH3, was performed as described in [62]. The chromogenic reaction was developed as described, using 0.45 mg/ml Nitroblue tetrazolium chloride (NBT; Roche) and 0.2 mg/ml 5-bromo-4-chloro-3-indolyl-phosphate (BCIP; Roche) in alkaline phosphatase buffer.

### TUNEL staining to detect apoptotic cells

Embryos were devitellinized, fixed in MEMFA, stained for β-gal and bleached. They were washed in 1× Terminal Deoxynucleotidyl Transferase (TdT) buffer (Invitrogen, Paisley, UK); 100 mM potassium cacodylate pH 7.2, 2 mM CoCl_2 _and 0.2 mM dithiothreitol) and incubated overnight at room temperature in TdT buffer with 0.5 μM alkaline stable digoxigenin-11-dUTP (Roche) and 150 U/ml recombinant TdT (Invitrogen). The TdT reaction was stopped by washing embryos at 65°C with PBST (phosphate-buffered saline, 0,1% Tween 20)/1 mM EDTA. Overnight incubation of the embryos in PBST/20% heat inactivated goat serum with anti-digoxigenin was performed at 4°C. Embryos were washed with PBS, and staining was developed using NBT/BCIP.

### Analysis of melanocyte distribution

Embryos were injected with 0.5 ng of xCdc4ΔFbox, xCdc4 or control GFP mRNA into each of the two animal dorsal cells of four-cell-stage embryos. At stage 43, the embryos were fixed in MEMFA and dehydrated overnight in ethanol. For the examination of melanocytes, individual embryos were photographed, the anterior region selected and the number of melanocytes counted using Image J software. For each condition, the number of melanocytes was averaged, the mean ± SEM was calculated and a Student's *t*-test performed.

### Western blotting

Embryos were lysed in 100 mM NaCl, 5 mM EDTA, 0.1% Triton X-100 and 50 mM β-glycerophosphate. Cleared supernatants were mixed with an equal volume of 2× SDS gel loading buffer (100 mM Tris pH 6.8, 4% SDS, 20% glycerol and 0.2% bromophenol blue), and dithiothreitol was added to a final concentration of 100 mM. One embryo equivalent per lane was loaded. In order to quantify protein levels, blots were imaged by using infrared fluorescence of appropriately tagged secondary antibodies and quantified by using a LiCOR Biosciences (Lincoln, Nebraska, USA) Odyssey scanner and software.

## Abbreviations

GFP: green fluorescent protein; ISH: *in situ *hybridization; PBS: phosphate-buffered saline; phH3: phosphorylated histone H3; Ppa: Partner of paired; RING: Really Interesting New Gene; SCF: Skp1-Cullin1-F-box; SEM: standard error of the mean; TdT: Terminal Deoxynucleotidyl Transferase; TUNEL: terminal deoxynucleotidyltransferase-mediated dUTP-biotin nick end labeling.

## Competing interests

The authors declare that they have no competing interests.

## Authors' contributions

ADA and HMW are joint first co-authors.

AP conceived the study, participated in its experimental design and coordination, and helped draft and refine the manuscript. HMW participated in study design, designed and undertook experiments and produced the first draft of the manuscript. HMW, MKS and RSH contributed to generation of new reagents and coordinated the collaboration. ADA designed and undertook the experiments during the revision of the manuscript. AP, HMW, ADA, CJH, MKS and RSH helped refine the manuscript. HMW was the sole contributor to Figures 5, 6, 7, and 12 and Additional files 2, 3, 4, 5 and 6, and the primary contributor to Figure 8. ADA was the primary contributor to Figures 2, 3, 4, 9, 10 and 11. HMW and ADA contributed to Figure 1. MKS was the primary contributor of Additional file 1. ADA and CJH contributed to Figure 8. HMW, ADA, CJH, RSH and AP analyzed and interpreted the data. All authors read and approved the final manuscript.

## Supplementary Material

Additional file 1 **Alignment of *X. laevis *Cdc4 isoforms to hCdc4α and hCdc4β**. ClustalW alignment of human (HU) and *X. laevis *(Xl) Cdc4α and Cdc4β. Xl Cdc4α shows 88% identity to HU Cdc4α. Similarly, Xl Cdc4β shows 86% identity to HU Cdc4β. Black boxes denote nuclear localization signals for Xl and HU Cdc4α; red box indicates F-Box motif; green boxes outline WD40 repeat region. Domain assignments were made using the Pfam program at the Sanger Centre [64]. The putative dimerization domain of Cdc4 (based on [64-66] is shown as a black dashed line. Grey regions indicate identity while blue signifies similar amino acids. A single point mutation was introduced into xCdc4α (nucleotide G1946A in the coding sequence, resulting in G649D in the protein) to introduce an Asp residue (denoted by red text at position 649) that is conserved amongst vertebrates, to correct what was most likely a cloning error. Click here for file

Additional file 2 **xCdc4ΔFbox does not affect anterior-posterior axis patterning**. We injected 1 ng of xCdc4ΔFbox (A,B,E,F,I,J) or xCdc4 mRNA (C,G,K) into one cell of two-cell-stage embryos; 1 ng of GFP mRNA (D,H,L) was injected as a control and β-gal mRNA was co-injected as a lineage tracer. Stage 16 to 18 embryos were stained for the forebrain/anterior midbrain marker *Otx2 *(A-D), the midbrain/hindbrain junction marker *En2 *(E-H), or the hindbrain marker *Krox20 *(I-L). Representative embryos from three independent pooled experiments are shown (n = 38 to 79; anterior view, dorsal up, injected side right). Numbers represent the percentages of embryos displaying each phenotype and white arrows highlight differences in expression of the markers on the injected side. Click here for file

Additional file 3 **xCdc4ΔFbox does not affect development of the myotome, the epidermis or primary neurons**. xCdc4ΔFbox, xCdc4 or control GFP mRNA (1 ng) was injected into one cell of two-cell-stage embryos. β-gal was co-injected as a lineage tracer. ISH was performed on stage 13 to 15 embryos for *MyoD *(A-C) and *epidermal keratin *(*EK*) (G-I), on stage 15 embryos for *neural β-tubulin *(*NβT*) (J-L) and at stage 18 to 20 for *heavy chain myosin *(*HCM*) (D-F). Representative embryos for the indicated injection are shown (dorsal view, anterior up, injected side on the right). Numbers are the percentage of embryos with normal phenotypes (n = 59 to 109). Click here for file

Additional file 4 **The F-box protein xSkp2 does not affect neural crest development**. xSkp2 or xSkp2ΔFbox mRNA (2 ng) was injected into one cell of two-cell-stage embryos. GFP mRNA (2 ng) was injected as a control. β-gal mRNA was co-injected as a lineage tracer. ISH for *Snail2 *was performed on stage 18 embryos. As an additional control, ISH was performed in parallel for *NβT *on stage 15 embryos. (A-C) Representative embryos (anterior view, dorsal up, injected side on the right) from *Snail2 *ISH for each injection. Numbers are the percentage of embryos displaying each phenotype (pooled data from two experiments; n = 53 to 61). (D-F) Representative embryos (dorsal view, anterior up, injected side on the right) injected with the indicated mRNA, from *NβT *ISH. Numbers are the percentage of embryos displaying each phenotype (n = 49 to 52). White arrows indicate reduced primary neurons on the injected side of the embryo. Click here for file

Additional file 5 **xCdc4ΔFbox does not affect placode development**. xCdc4ΔFbox, xCdc4 or control GFP mRNA (1 ng) was injected into one cell of two-cell-stage embryos. β-gal was co-injected as a lineage tracer. ISH was performed on stage 16 to 18 embryos for the placodal marker *Six1 *(n = 49 to 86). (A-C) Representative embryos are shown for the indicated injections (dorsal view, anterior up, injected side right). Numbers represent the percentage of normal embryos. Click here for file

Additional file 6 **c-Jun is a negative regulator of neural crest development**. c-Jun mRNA (0.5 ng or 1 ng) was injected into one cell of two-cell-stage embryos. GFP mRNA was injected as a control, and β-gal mRNA was injected as a lineage tracer (light blue unilateral staining). Whole mount ISH was performed for *Snail2 *(A-C) or *c-Myc *(D-F) expression. The area of *Snail2 *staining on the injected side was expressed as a ratio of the area on the uninjected side. The mean ± SEM ratio (n = 37 to 87) is shown for each injection. Representative embryos are shown (anterior view, dorsal up, injected side right). The average percentage reduction in *Snail2 *staining on the injected side, compared to embryos injected with GFP, is shown for *Snail2 *ISHs. For *c-Myc *ISHs, the percentage of embryos showing the phenotype is displayed. Click here for file
