# Supplementary figures and images for: The F-box protein Cdc4/Fbxw7 is a novel regulator of neural crest development in Xenopus laevis
Source: Neural Dev. 2010 Jan 4;5:1. doi: 10.1186/1749-8104-5-1 (PMC2819241; doi:10.1186/1749-8104-5-1)

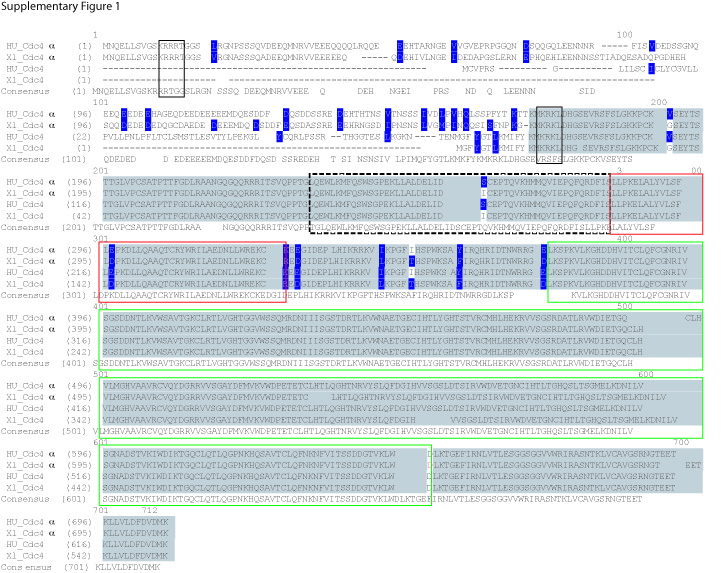

Supplement: Additional file 1 — Alignment of X. laevis Cdc4 isoforms to hCdc4α and hCdc4β. ClustalW alignment of human (HU) and X. laevis (Xl) Cdc4α and Cdc4β. Xl Cdc4α shows 88% identity to HU Cdc4α. Similarly, Xl Cdc4β shows 86% identity to HU Cdc4β. Black boxes denote nuclear localization signals for Xl and HU Cdc4α; red box indicates F-Box motif; green boxes outline WD40 repeat region. Domain assignments were made using the Pfam program at the Sanger Centre [64]. The putative dimerization domain of Cdc4 (based on [64-66] is shown as a black dashed line. Grey regions indicate identity while blue signifies similar amino acids. A single point mutation was introduced into xCdc4α (nucleotide G1946A in the coding sequence, resulting in G649D in the protein) to introduce an Asp residue (denoted by red text at position 649) that is conserved amongst vertebrates, to correct what was most likely a cloning error. [file 1749-8104-5-1-S1.JPEG]

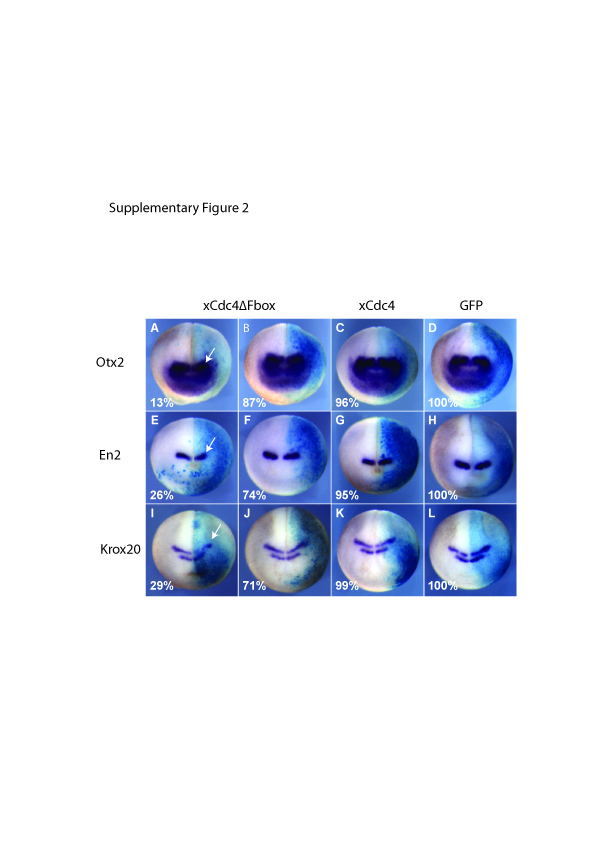

Supplement: Additional file 2 — xCdc4ΔFbox does not affect anterior-posterior axis patterning. We injected 1 ng of xCdc4ΔFbox (A,B,E,F,I,J) or xCdc4 mRNA (C,G,K) into one cell of two-cell-stage embryos; 1 ng of GFP mRNA (D,H,L) was injected as a control and β-gal mRNA was co-injected as a lineage tracer. Stage 16 to 18 embryos were stained for the forebrain/anterior midbrain marker Otx2 (A-D), the midbrain/hindbrain junction marker En2 (E-H), or the hindbrain marker Krox20 (I-L). Representative embryos from three independent pooled experiments are shown (n = 38 to 79; anterior view, dorsal up, injected side right). Numbers represent the percentages of embryos displaying each phenotype and white arrows highlight differences in expression of the markers on the injected side. [file 1749-8104-5-1-S2.JPEG]

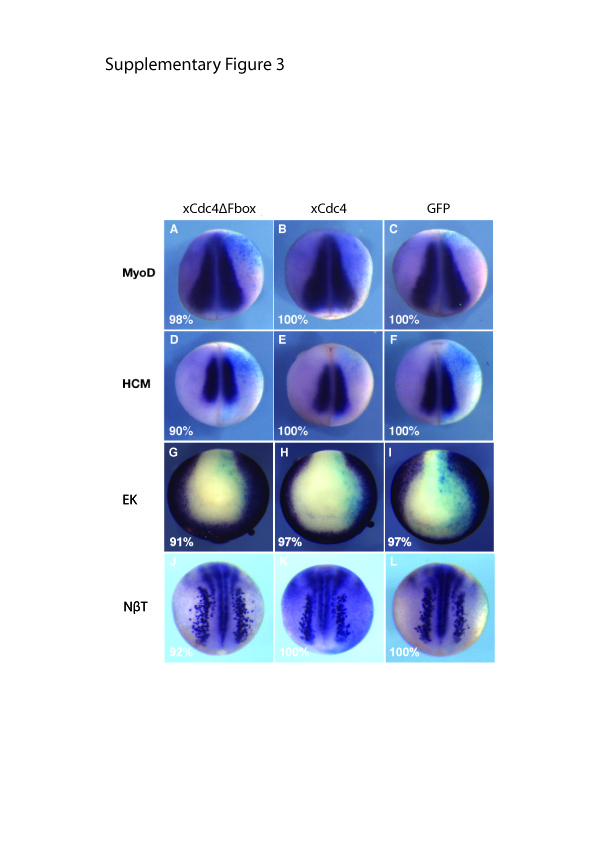

Supplement: Additional file 3 — xCdc4ΔFbox does not affect development of the myotome, the epidermis or primary neurons. xCdc4ΔFbox, xCdc4 or control GFP mRNA (1 ng) was injected into one cell of two-cell-stage embryos. β-gal was co-injected as a lineage tracer. ISH was performed on stage 13 to 15 embryos for MyoD (A-C) and epidermal keratin (EK) (G-I), on stage 15 embryos for neural β-tubulin (NβT) (J-L) and at stage 18 to 20 for heavy chain myosin (HCM) (D-F). Representative embryos for the indicated injection are shown (dorsal view, anterior up, injected side on the right). Numbers are the percentage of embryos with normal phenotypes (n = 59 to 109). [file 1749-8104-5-1-S3.JPEG]

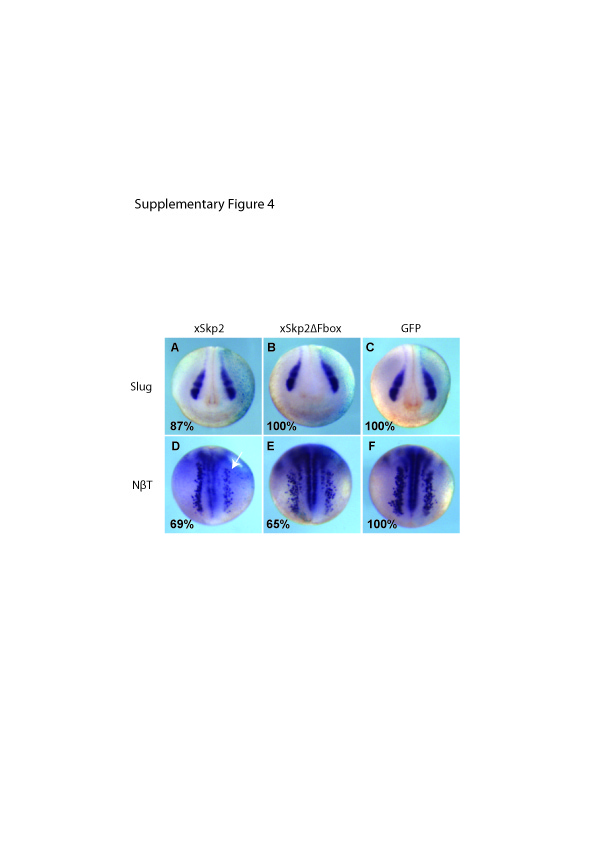

Supplement: Additional file 4 — The F-box protein xSkp2 does not affect neural crest development. xSkp2 or xSkp2ΔFbox mRNA (2 ng) was injected into one cell of two-cell-stage embryos. GFP mRNA (2 ng) was injected as a control. β-gal mRNA was co-injected as a lineage tracer. ISH for Snail2 was performed on stage 18 embryos. As an additional control, ISH was performed in parallel for NβT on stage 15 embryos. (A-C) Representative embryos (anterior view, dorsal up, injected side on the right) from Snail2 ISH for each injection. Numbers are the percentage of embryos displaying each phenotype (pooled data from two experiments; n = 53 to 61). (D-F) Representative embryos (dorsal view, anterior up, injected side on the right) injected with the indicated mRNA, from NβT ISH. Numbers are the percentage of embryos displaying each phenotype (n = 49 to 52). White arrows indicate reduced primary neurons on the injected side of the embryo. [file 1749-8104-5-1-S4.JPEG]

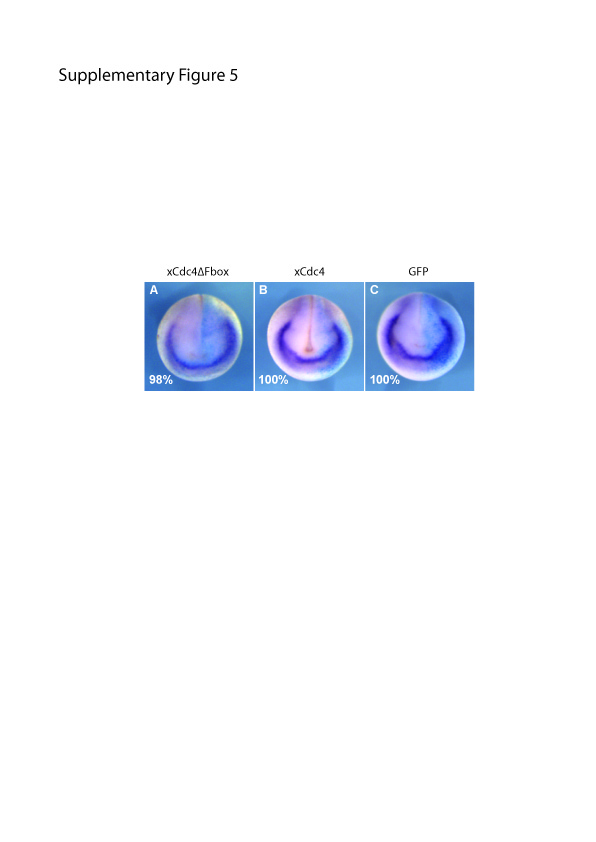

Supplement: Additional file 5 — xCdc4ΔFbox does not affect placode development. xCdc4ΔFbox, xCdc4 or control GFP mRNA (1 ng) was injected into one cell of two-cell-stage embryos. β-gal was co-injected as a lineage tracer. ISH was performed on stage 16 to 18 embryos for the placodal marker Six1 (n = 49 to 86). (A-C) Representative embryos are shown for the indicated injections (dorsal view, anterior up, injected side right). Numbers represent the percentage of normal embryos. [file 1749-8104-5-1-S5.JPEG]

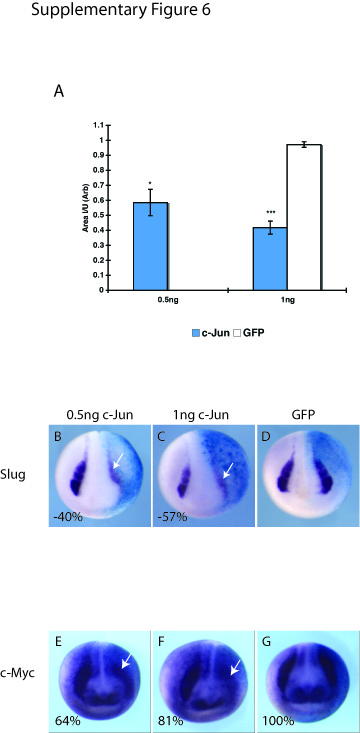

Supplement: Additional file 6 — c-Jun is a negative regulator of neural crest development. c-Jun mRNA (0.5 ng or 1 ng) was injected into one cell of two-cell-stage embryos. GFP mRNA was injected as a control, and β-gal mRNA was injected as a lineage tracer (light blue unilateral staining). Whole mount ISH was performed for Snail2 (A-C) or c-Myc (D-F) expression. The area of Snail2 staining on the injected side was expressed as a ratio of the area on the uninjected side. The mean ± SEM ratio (n = 37 to 87) is shown for each injection. Representative embryos are shown (anterior view, dorsal up, injected side right). The average percentage reduction in Snail2 staining on the injected side, compared to embryos injected with GFP, is shown for Snail2 ISHs. For c-Myc ISHs, the percentage of embryos showing the phenotype is displayed. [file 1749-8104-5-1-S6.JPEG]
